# Escalating competition in changing seas: baleen whales exploit killer whale prey herding

**DOI:** 10.1098/rstb.2025.0197

**Published:** 2026-10-15

**Authors:** Eve Jourdain, Chérine Baumgartner, David Cade, Paolo Domenici

**Affiliations:** ^1^ Norwegian Orca Survey, Andenes, Norway; ^2^ Department of Environmental Systems Science, Swiss Federal Institute of Technology Zurich, Zürich, Switzerland; ^3^ Orcestra, Zurich, Switzerland; ^4^ Hopkins Marine Station, Stanford University, Stanford, CA, USA; ^5^ IBF, CNR, Institute of Biophysics, Pisa, Italy

**Keywords:** predator, interspecific competition, kleptoparasitism, foraging ecology

## Abstract

Climate change and other anthropogenic pressures are rapidly reshaping marine ecosystems, altering prey availability and predator interactions. Yet empirical evidence of how these relationships shift under changing conditions remains scarce. Drawing on over a decade of monitoring in a subarctic fjord system, we provide a rare insight into these dynamics. In northern Norway, the winter aggregation of herring (*Clupea harengus*) and their primary predator, the killer whale (*Orcinus orca*), has shifted northward under the combined influence of climate change and fishing pressure. This redistribution has brought baleen whales into the same foraging grounds, leading to direct interference competition. Humpback (*Megaptera novaeangliae*) and fin whales (*Balaenoptera physalus*) frequently intrude into killer whale carousel feeding events, exploiting dense, shallow prey patches optimized for lunge feeding. Such intrusions often end hunts prematurely, imposing energetic costs on killer whales. Meanwhile, the rapid growth of whale-watching tourism in these fjords further disrupts foraging activity. Integrating unpublished data and existing literature, this article highlights the asymmetric energetic costs and benefits arising from these emerging interactions; processes likely widespread, yet still largely undetected in other marine ecosystems.

This article is part of the theme issue ‘Multi-predator feeding aggregations in pelagic waters’.

## 1. Introduction

Marine ecosystems are experiencing large-scale transformations as climate change and human exploitation reshape the physical and biological architecture of the ocean. Rising sea temperatures, ocean acidification, deoxygenation and shifting current patterns are redefining the habitats available to marine life [1,2]. Species respond to environmental change by tracking their preferred thermal and trophic niches, shifting in space and time to maintain favourable conditions [1]. Resulting range shifts are now recognized as one of the most consistent biological consequences of ocean warming [3]. In higher latitudes, many species have expanded their ranges or migrated poleward, following cooler waters and productive feeding zones [4]. Such movements rarely occur uniformly across trophic levels [5]. As a result, predators and prey that once coincided may become separated, while species that historically occupied different habitats can newly overlap. At the same time, industrial fisheries extract enormous biomass, redistribute year classes and disrupt food webs, further altering the structure of marine communities [6]. The combined effects of climate- and other anthropogenic-driven change are thus not limited to redistributions of species but extend to a reorganization of how they interact across the pelagic realm.

As species migrate, asymmetrical costs and benefits among interacting species may arise. More mobile predators may track shifting prey or favourable habitats more effectively, gaining energetic and fitness advantages, whereas less mobile species may experience reduced access to resources [1,5]. In new habitats, opportunistic species may innovate [7], learn from others [8] or engage in kleptoparasitism, i.e. stealing prey from other species [9]. The latter creates situations where one species gains enhanced feeding opportunities without reciprocation, effectively *parasitizing* the efforts of others, which in turn bear disproportionate energetic costs [10]. These altered interactions can exacerbate physiological stress, thereby synergistically magnifying responses to abiotic pressures and producing impacts greater than the sum of individual stressors [11,12]. Consequently, even species initially resilient—perhaps because their prey remain accessible, or they can effectively track shifting resources—may eventually be affected as changing ecological interactions can intensify abiotic stressors [12]. Recognizing such uneven outcomes is therefore essential for predicting population and ecosystem responses, as the effects of climate change are rarely uniform and depend strongly on the ecological context of interacting species. Neglecting these dynamics risks underestimating exposure and sensitivity to change, particularly for specialists whose persistence relies on access to specific resources. Despite broad recognition of the importance of ecological interactions, empirical documentation of their shifts in changing habitats and cascading effects remains scarce.

This study takes advantage of a subarctic fjord system monitored for over a decade that offers a rare empirical window into these processes. In the Norwegian Sea and its adjacent fjords, shifting distributions of the Norwegian spring-spawning stock of the Atlantic herring (*Clupea harengus*, hereafter NSS herring; [13,14]) have reorganized predator assemblages, bringing together species that historically foraged separately. Using this uniquely accessible and long-studied system, we examine how climate- and other anthropogenic-driven prey redistributions reshaped cetacean interspecific interactions over surprisingly short ecological timescales. In addition, we consider the rapid growth of whale-watching activities in these fjords as an intensifying anthropogenic component of the system [15]. This expanding tourism industry, owing to the increased accessibility and predictability of whale aggregations, introduces additional pressures that may further influence predator behaviour, habitat use, energy budgets and physiological stress. Because the species involved are well studied, this setting allows us to conceptually identify the likely mechanisms and consequences underlying the observed trends, providing insights into processes that are presumably widespread but remain undetected in less accessible marine ecosystems.

## 2. Prey shifts redefine predator interactions

The NSS herring, one of the world's largest fish stocks, has long structured both marine food webs and human exploitation in the Northeast Atlantic [16]. Its seasonal movements, i.e. spawning, feeding and wintering, are associated with predictable variations in school density, fat content and behaviour, governing its profitability to its predators [17–19]. Wintering aggregations in particular have been highly profitable when herring form their densest aggregations and reach their highest fat content [19,20]. Over time, the location of these seasonal grounds has shifted in response to variability in climatic and oceanographic conditions and fishing pressure [14,16,21]. Following the near-collapse of the stock in the late 1960s, wintering areas moved from midway between Iceland and Norway towards the northern Norwegian fjords [16]. This shift established a highly profitable feeding ground in the Tysfjord region, where killer whales exploited dense, lipid-rich herring schools for more than two decades—with little competition beyond fish predators, as baleen whales were completely absent ([22–26], figure 1A). In the mid-2000s, herring again altered their wintering behaviour, moving farther north into more pelagic regions [13]. This redistribution triggered a cascade of predator responses: humpback (*Megaptera novaeangliae*) and fin whales (*Balaenoptera physalus*), previously absent from historical herring wintering grounds (Tysfjord, figure 1A), increasingly) appeared at these new herring sites, forming unprecedented mixed-species aggregations [22,27].

**Figure 1.**
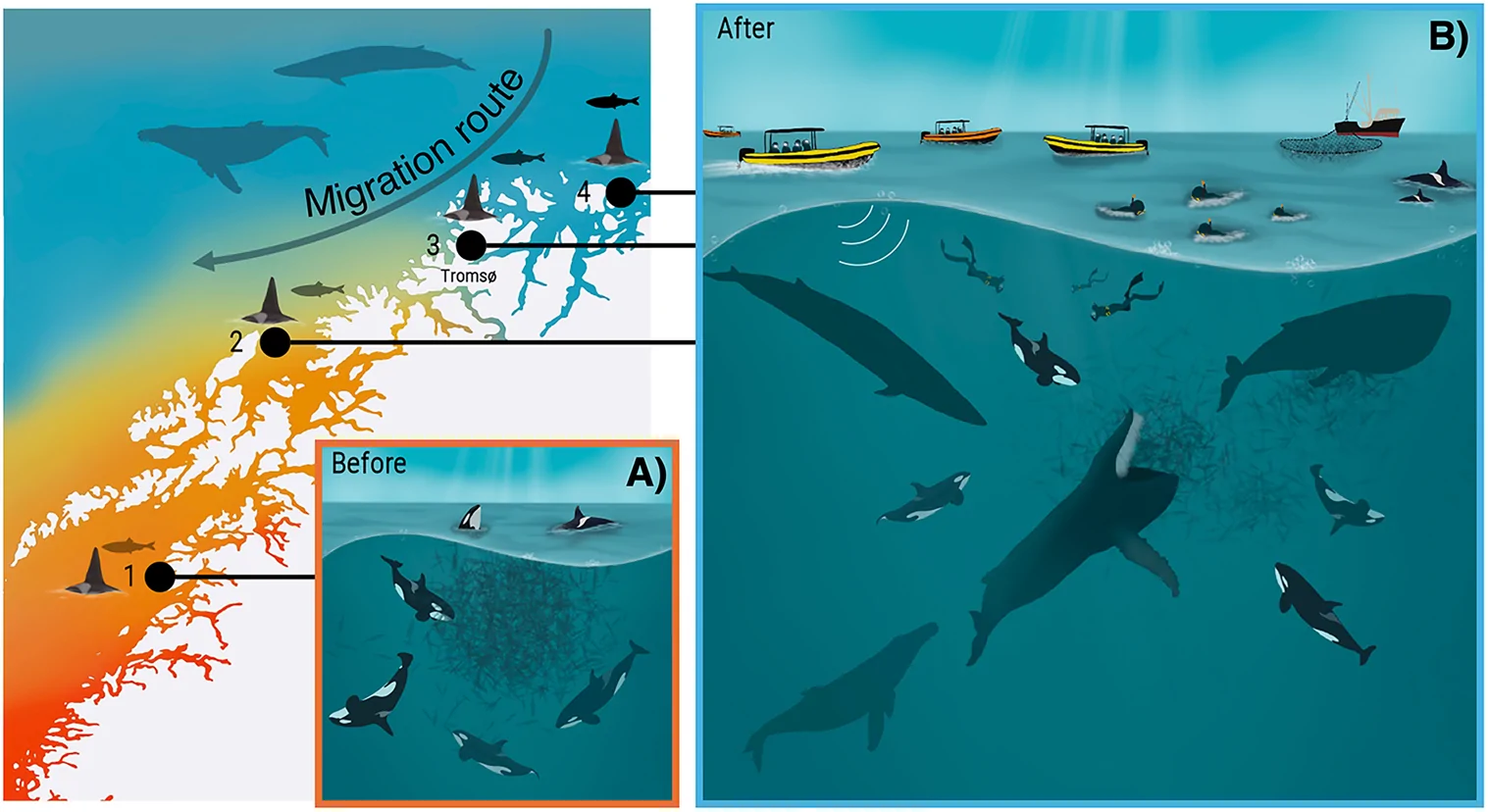
Infographic illustrating how the northward shift of Norwegian Spring-Spawning herring wintering grounds, driven by climate change and fishing pressure, has reshaped ecological interactions and intensified tourism pressure. (A) ‘Before’ depicts killer whale carousel feeding with little to no disturbance, owing to baleen whales being totally absent, when herring wintered in the Tysfjord region (location 1); (B) ‘After’ illustrates the increasingly common situation in which carousel feeding is disrupted by intruding baleen whales and a growing whale-watching fleet, promoted by the progressive northward shift of herring wintering grounds (from location 1, to 2, 3 and 4.

Off Norway, both humpback and fin whales are opportunistic feeders, shifting among euphausiids, capelin and small schooling fish depending on prey availability [28–31]. Historically, their numbers were relatively stable, and their distributions closely tracked macrozooplankton abundance [32]. However, a prolonged decline in macrozooplankton biomass likely contributed to the marked distributional changes observed from the early 2000s onward [33]. Humpback whale abundance increased substantially (from approx. 1000, coefficient of variation (CV) = 0.25, confidence interval (CI): 650–1700 in 1995, see [34]; to approx. 10 000, CV = 0.38, 95% CI: 4900–23 000 in 2014–2018, see [35]) in the Barents and Norwegian Seas, where their occurrence became increasingly linked to NSS herring [32,35,36]. Between 2009 and 2012, coinciding with historically high biomasses of NSS herring and other pelagic fish, fin whales were increasingly observed feeding on fish, notably herring and capelin [30]. From 2013 to 2018, both humpback and fin whales exhibited pronounced northward shifts in their summer distributions, once again closely correlated with NSS herring occurrence [28,30]. While these patterns underscore the importance of herring as a key prey resource, the redistribution of the NSS herring stock effectively positioned wintering grounds within direct reach of migrating baleen whales travelling between northern summer feeding areas and southern breeding grounds [13,27,32].

The northward redistribution of herring and their predators has drawn increasing human attention. The predictable presence of large whale aggregations, now concentrated near Tromsø, the logistical hub of northern Norway, has enabled rapid growth in whale-watching and snorkelling activities over the past decade, operating entirely without regulation [15]. These developments introduced new anthropogenic pressures and interactions within the evolving predator assemblages. Together, these dynamics illustrate how prey shifts can cascade through trophic levels, reshaping predator distributions, interactions and human engagement with the ecosystem.

## 3. Uneven rewards among predators

Carousel feeding is a sequential hunting process where killer whales expend initial energy to create conditions for a highly efficient prey capture phase. In winter, NSS herring aggregate in dense layers at depths of 150–350 m, presumably to reduce predation by visually oriented predators [20,37], though smaller schools may be found at shallower depths [38]. To access the prey, killer whales dive deep down to 150 m to isolate and force a portion of the school (i.e. up to 1000 tons of fish) towards the surface [29,39]. These manoeuvres are performed cooperatively, typically involving 10–20 individuals or more that work together to herd the school into a tight formation; a process that requires precise coordination and complex social interactions [26,29,39,40]. This initial phase represents an energetic investment for an uncertain outcome, as attempts can fail.

However, this upfront effort yields advantages once the herring are brought near the surface. First, the surface acts as a physical barrier, limiting the prey's escape routes. Second, killer whales can use the reflection of available light on their white undersides to ‘flash’ the herring, scaring them into increasingly dense schools [24,26,40,41]. This compact formation maximizes the efficiency of their primary capture method, i.e. stunning fish with underwater tail slaps, since denser herring schools allow more prey to be struck with a single slap [26,40,41]. Restricting the hunt to shallow water further conserves energy by allowing short dives and constant oxygen access to sustain high metabolic output [37]. Although carousel feeding requires considerable effort in its initial phase, it is highly rewarding in terms of prey capture rates, provided other predators do not interfere [42].

After herring shifted their wintering grounds to pelagic areas in 2009, humpback whales newly appeared and have, ever since, been observed exploiting ongoing killer whale carousel feeding events ([22]; figures 1B and 2; supplementary material, table S1). In recent years, fin whales have also been increasingly recorded joining these feeding bouts (figures 2 and 3A; supplementary material, table S1). Both species typically enter once the herring ball is fully formed, gaining access to a dense, shallow prey patch optimally configured for lunge feeding [43]. Indeed, killer whales were identified as the catalyst species, with baleen whales arriving later, in 99% of carousel feeding events fully documented from start to finish between 2013 and 2024 ([22]; supplementary material, table S1). Baleen whales typically appear in small groups of one to three individuals, lunging one or several times through the herring school [22]. These intrusions led to the immediate termination of hunts in 49% of the 71 fully documented events (supplementary material, table S1). Such early terminations likely occur because the herring ball becomes partially consumed or fragmented. In northern Norwegian fjords, herring schools typically range from approximately 5 to 20 m in diameter ([26], E. Jourdain 2014–2025, unpublished data; corresponding to approx. 65–4200 m³ in volume). Consequently, an entire bait ball cannot be consumed in a single lunge; for example, a 12 m humpback whale has an estimated engulfment volume of only approximately 22 m³ [44]. Even for prey patches comparable in size to a whale's gulp, kinematic models indicate that approximately 70% of the fish escape during a lunge [45]. This suggests that disruption of school cohesion, or interference with the killer whales’ coordinated hunting tactics [26], is likely a more important driver of foraging cessation than complete prey consumption. Regardless, energetic gain for killer whales seems unlikely unless baleen whale lunges also stun or disorient herring, similar to sea lions in California that opportunistically feed on anchovies dislodged by lunge-feeding whales [46]. In Norway, the observations that feeding events often terminate with killer whales immediately leaving the site after baleen whale intrusions point to energetic losses for killer whales, as the energy already invested in deep dives, herding and surface manipulation of herring schools yields little or no return.

**Figure 2.**
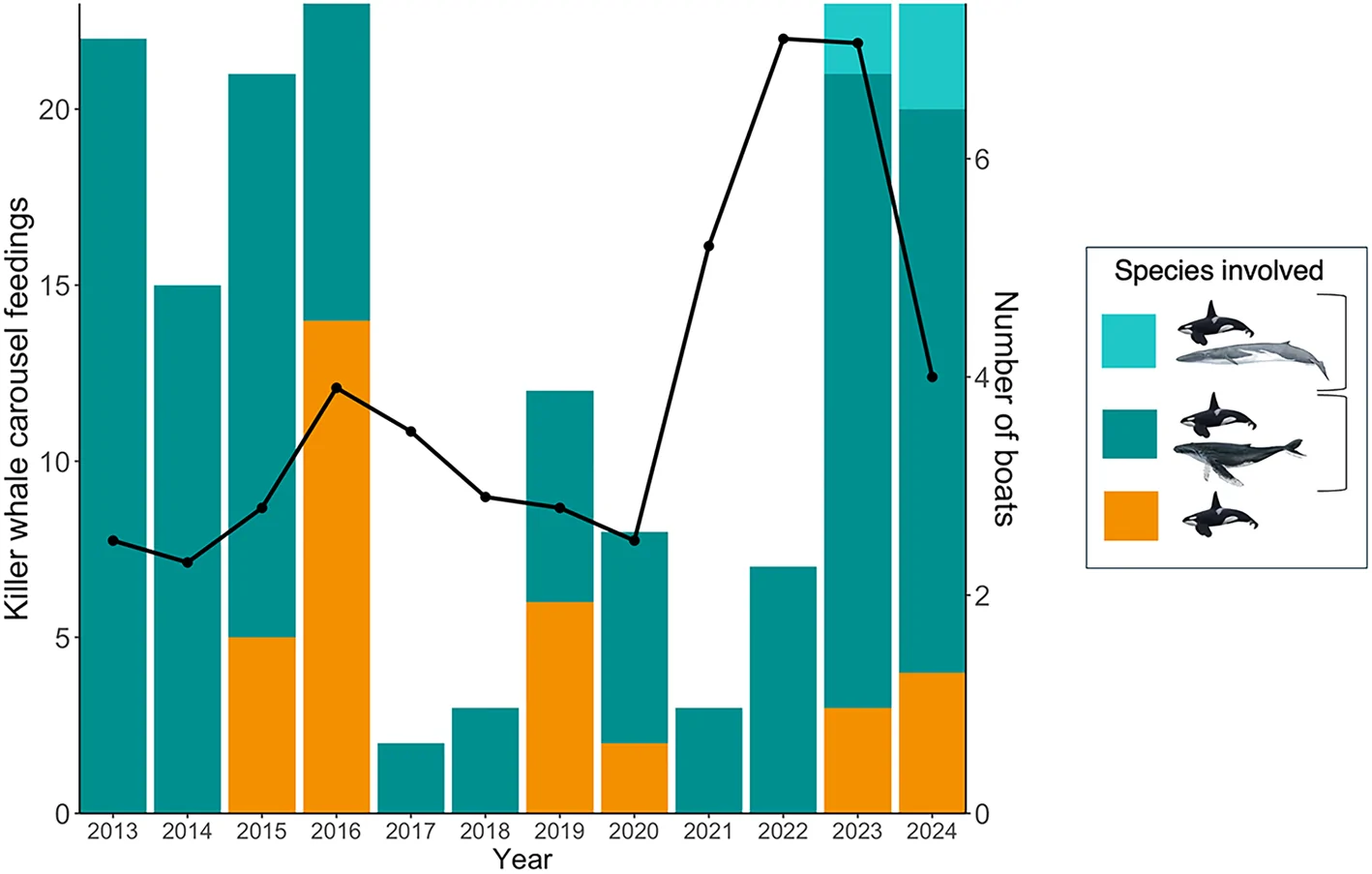
Number of killer whale carousel feeding events documented each November at herring wintering grounds from 2013 to 2024. Colours distinguish events involving killer whales only (orange) from those where humpback (dark teal) and fin whales (light teal) joined to lunge feed. The mean number of whale-watching boats present (line plot, secondary *y* axis) during killer whale carousel feeding events is also shown. Overall, 157 carousel feeding events were recorded in 2013–2024, of which 128 involved baleen whales.

**Figure 3.**
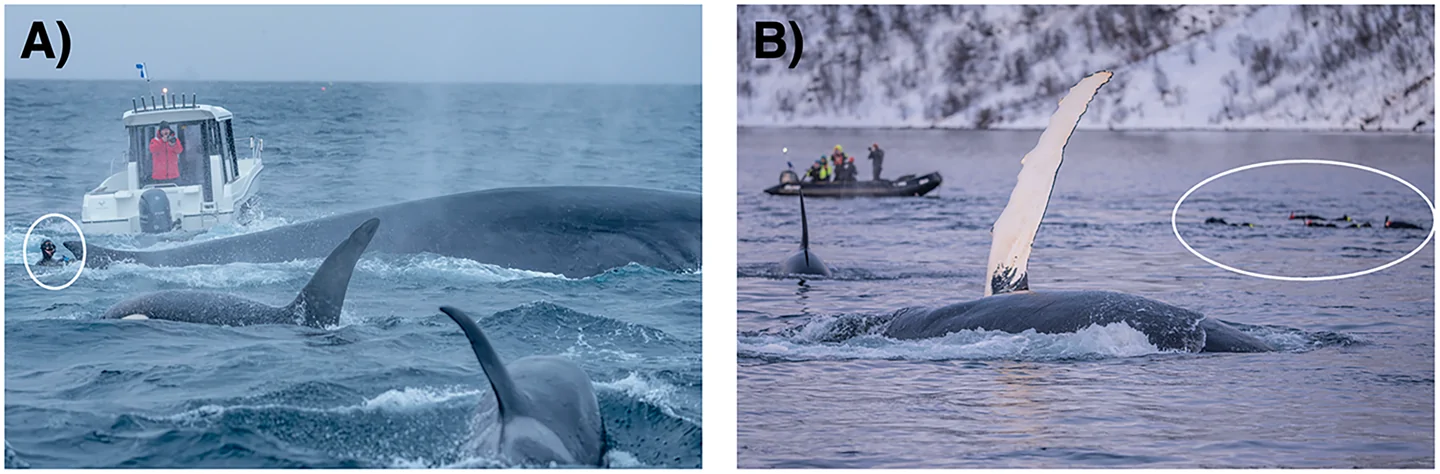
(A) Killer whale carousel feeding disrupted by the intrusion of a lunging fin whale in November 2023, alongside interference from boats and divers. (B) One of the 19 documented incidents showing killer whales harassing humpback whales after the latter abruptly intruded and lunged through their herring bait ball. In this image, the humpback whale keeps its pectoral flipper elevated, likely in a defensive posture to avoid being bitten by the orcas. In both events, whale-watching vessels and snorkellers simultaneously interfere on the feeding aggregation, causing additional disturbance through their close proximity to the animals. Photo credits: Eve Jourdain.

Both humpback and fin whales face high energetic demands associated with lunge feeding, which requires rapid acceleration, engulfing large volumes of water and repeated mouth openings [47–49]. Effective foraging, therefore, depends on prey being aggregated into dense, compact patches of sufficient size and shape that maximize prey capture per lunge while reducing effort [45]. By feeding on herring balls already densely herded by killer whales, baleen whales can access prey with substantially lower energy expenditure. Moreover, the presence of killer whales may further decrease the likelihood of fish escaping, enhancing feeding efficiency for the baleen whales. This creates a pronounced asymmetry in foraging costs as killer whales bear the energetic burden of corralling the herring and baleen whales capitalize on the prey with little effort, representing a clear case of interspecific kleptoparasitism.

## 4. Cues and mechanisms

The viability of this kleptoparasitic strategy hinges on the baleen whales’ ability to detect feeding events across a vast habitat. In the field, we commonly observe baleen whales approaching killer whale feeding events from afar, at high speeds. Indeed, killer whale carousel feedings provide several acoustic cues that overlap with the hearing sensitivity of humpback whales (0.7–10 kHz, [50]). Signals falling directly within humpback whales’ hearing range include herring gas-release sounds (3–7 kHz, [51]), which the fish produce under predation, and the lower-frequency components of killer whale pulsed calls (0.5–120 kHz, [52,53]). Pulsed calls are produced at high rates during foraging, likely to facilitate group coordination [26,53–57]. Other broadband signals, though centred in the ultrasonic range, also produce energy that is potentially detectable. Killer whale tail slaps, with a mean centre frequency of approximately 46.1 kHz, are broadband pulses with frequency components recorded from near 0 kHz to at least 150 kHz [58]. Similarly, echolocation clicks (centre frequency range 22–49 kHz) are broadband signals that possess a distinct low-frequency peak at 24 kHz [55]. While this peak is well above humpback whales’ optimal hearing range, recent evidence suggests the upper physiological limit of these whales’ hearing extends to higher frequencies than previously thought (up to at least 16 kHz, [59]). While some of these signals are quieter than others, and absolute detection range varies with source levels, ambient noise and propagation, humpback whales are known to detect at least killer whale vocalizations over long distances [60]. Therefore, eavesdropping on carousel-feeding killer whales provides baleen whales with cues to locate ongoing feeding events; a ‘dinner bell’ effect confirmed by playback experiments that elicited directional approaches towards broadcast sounds of herring-feeding killer whales [61]. Beyond these sensory mechanisms, social learning among baleen whales likely facilitates the spread of knowledge about such foraging cues [8], enabling more individuals to exploit them and thereby intensifying interference competition over time.

## 5. Behavioural responses under shifting interspecific interactions

Interspecies interactions are not static and can drive changes in behaviour. Since 2016, killer whales have increasingly been observed harassing humpback whales at the wintering grounds, typically by attacking their appendages (figure 3B; supplementary material, table S1). These encounters are conspicuous, as humpbacks often respond by lifting their flukes or long pectoral fins above the sea surface away from the attackers. Because killer whales have never been observed preying on humpback whales in Norway, these events are unlikely to be predatory in nature and instead likely reflect agonistic interactions, potentially arising from irritation or frustration following repeated and almost systematic loss of access to prey during feeding events. Aggressive behaviours may evolve in response to interference competition as an attempt to exclude competitors from limited resources [62–64].

Another way in which interference competition may drive behavioural change in killer whales is through shifts in foraging strategies that buffer against disturbance. Killer whales employ several herring-feeding techniques, among which carousel feeding appears to yield high foraging success in the absence of competitors [42]. However, interference from baleen whales has likely reduced its profitability. To mitigate energetic losses, killer whales may increasingly adopt alternative foraging behaviours that minimize competition [9,10]. These include scavenging on discards from herring purse-seiners, a behaviour that pre-dates the recent increase in baleen whale presence but may now serve as a competitive refuge [42,56,65]. Other strategies involve deep feeding dives that entail little or no herding of dense herring schools and therefore offer few benefits to baleen whales, reducing the likelihood of competitive intrusion [42]. Killer whales have also been observed hunting in shallow waters, where pairs cooperate in a division of roles: a ‘striker’ tail-slaps the prey while a ‘helper’ forms a physical barrier, herding the school towards the striker to maximize the number of stunned fish [66]. This strategy, observed in association with herring schools larger than 3000 m², remains unclear in origin, i.e. whether killer whales actively drove the herring into the shallows or exploited schools already aggregated there. Regardless, humpback whales have also been documented feeding in these shallow areas alongside killer whales (E. Jourdain 2014–2025, unpublished data), suggesting they may have learned to exploit the same opportunities. Over the study period, carousel feeding shows a possible declining trend despite stable research effort (figure 4), possibly reflecting fewer or shorter bouts caused by baleen whale presence and/or a strategic shift towards foraging modes that reduce competition and maximize energetic efficiency.

**Figure 4.**
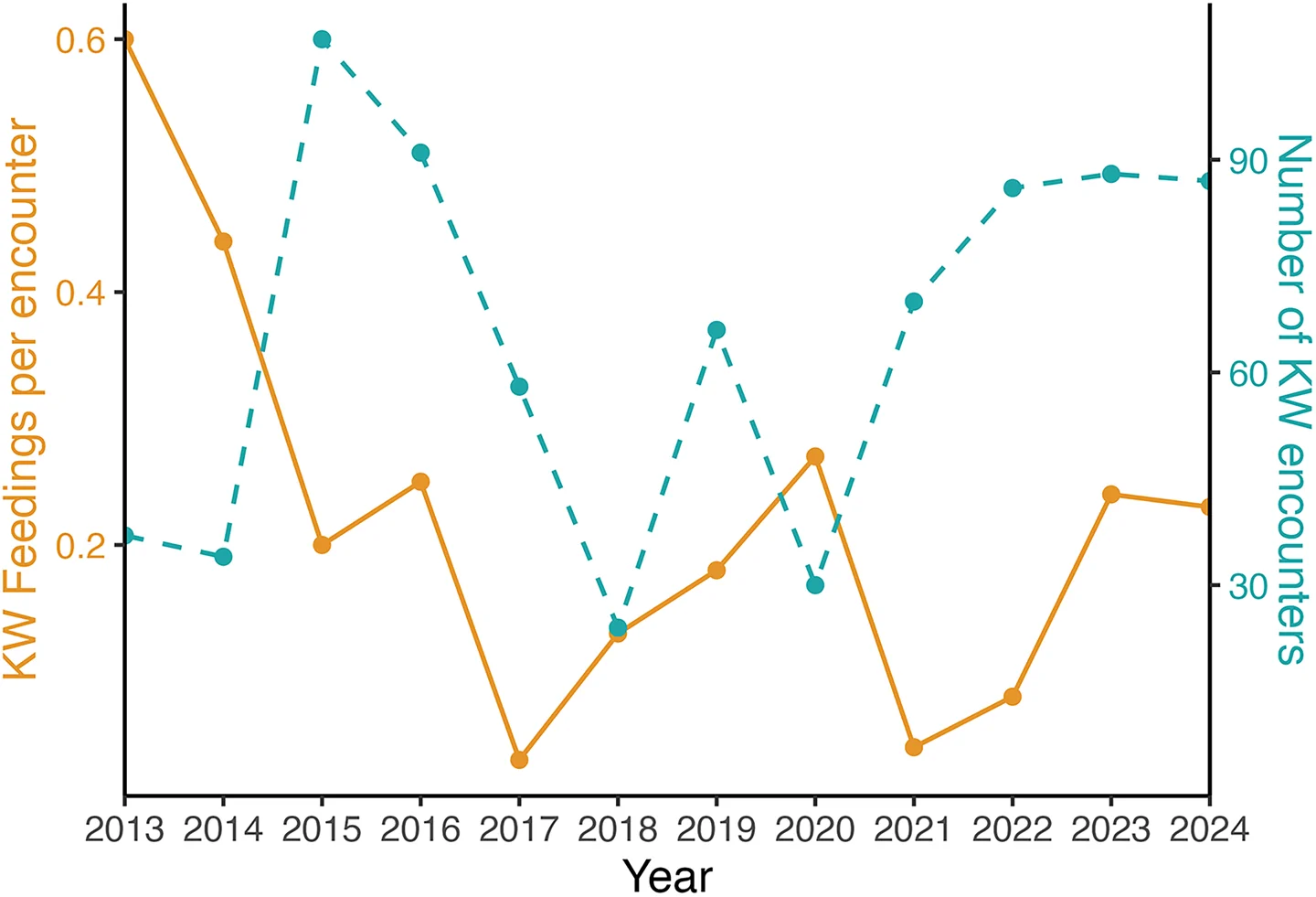
Number of killer whale (KW) carousel feeding events, standardized by the total number of killer whale encounters recorded at herring wintering grounds (orange line), showcasing the relatively stable effort in the field throughout the study period. Total annual number of killer whale encounters is also shown for reference (teal dashed line, secondary *y* axis).

Ubiquitous competitive interference may also act as an ecological pressure, driving shifts in signalling traits aimed at minimizing competitive interactions [67]. Anecdotal observations indicate that killer whales have become less vocally active during carousel feeding in recent years compared with historical descriptions, a pattern currently under investigation. Since acoustic cues allow eavesdropping baleen whales to detect and locate carousel-feeding events, reduced vocal output could represent an adaptive response aimed at lowering detectability and limiting kleptoparasitic intrusion. However, tail-slap sounds, which are integral to the feeding process, remain unavoidable and may continue to serve as a reliable cue for nearby competitors. Whether the audible frequencies of these tail slaps can be detected by baleen whales remains to be investigated.

## 6. Whale watching activities as a multiplier

These emerging ecological pressures on killer whales are further compounded by growing anthropogenic disturbance from the rapid expansion of whale-watching and in-water tourism within the same fjords ([15]; figure 2; supplementary material, table S1). Vessels and snorkellers now frequently converge on feeding aggregations, introducing additional disturbance coinciding with intense prey competition (figure 3). The cumulative impact is considerable: killer whales that have invested substantial energy into corralling herring are simultaneously exposed to intruding humpback and fin whales, and disruptive underwater noise as well as physical obstruction from vessels and swimmers (figures 1 and 3). The physical presence of vessels can force whales to modify their intended swimming paths within the coordinated carousel formation (visible in figure 3A), preventing them from manoeuvring with the intention and precision required to control the herring school. As a result, they may lose feeding potential normally gained from a well-executed carousel event. By increasing locomotion and vigilance, these disturbances may elevate energetic costs while simultaneously reducing prey intake.

Although winter days in northern Norway provide only 3–6 hours of daylight, leaving roughly 18–21 hours without tourism-driven disturbance, this does not necessarily translate into compensatory feeding opportunities. Herring naturally ascend towards the surface at night [17], which could, in theory, allow killer whales to forage at reduced energetic cost. However, tagging data from five Norwegian killer whales at herring wintering grounds in 2016–2017 indicated that individuals engage in alternative feeding behaviours during dark hours [40]. The apparent absence of carousel feeding during night-time remains inconclusive given the limited sample size. Interestingly, a neighbouring Icelandic herring-feeding population has been shown to forage at night using a similar encircling and tail-slap strategy, suggesting that nocturnal carousel feeding in Norway may have gone undetected to date [68]. Alternatively, since dark hours in northern Norwegian fjords coincide with peak herring fishing activity, killer whales may instead focus on scavenging around purse-seine nets during this time.

Acoustic disturbance adds another layer of impact, as vessel engines, propeller cavitation noise, along with sounds from in-water human activity can mask or interfere with the acoustic cues killer whales use for group coordination and prey localization [26,29,56]. Putland *et al.* [69] showed that vessel noise within just 10 km of whales can reduce the communication space of baleen whale species by up to 99% compared with natural ambient conditions. In Norway, where the whale-watching distance is unregulated, many vessels routinely approach whales at the same time within 20–50 m, or closer (figure 3). Thus, the acoustic environment around the whales is far more degraded, dominated by intense, overlapping vessel noise that likely masks biologically relevant signals, a scenario well documented in other killer whale populations [70,71]. This problem is further amplified by in-water tourism: by its very nature, snorkelling activities require boats to approach within metres of the whales so that participants can attempt underwater observations ([15]; figure 3). Such encounters inherently promote extremely close approaches and aggressive vessel manoeuvres to maintain proximity, thereby amplifying both physical and acoustic disturbance. To compensate, whales may need to increase their vocal output to maintain effective coordination, which in turn raises the likelihood of detection by baleen whales that can then locate and disrupt their feeding events [61,70,72]. Close approaches also introduce physical risks, including accidental strikes, while distraction caused by human presence may reduce vigilance towards competing species. Behavioural responses to disturbance may also shift, with some individuals becoming desensitized and tolerating unsafe proximity. Beyond responses to immediate encounters, years of repeated close approaches combined with continuous vessel traffic may have conditioned behavioural responses even to distant vessel noise. For example, simply hearing boats in the soundscape, even when they are not approaching or attempting close encounters, may lead to reduced resting times or increased swim speeds, imposing high energetic costs beyond the immediate disturbance events [73].

## 7. Contrasted population-level effects for the competitors

Cetaceans typically store 25–50% of their body mass as blubber, which plays key roles in thermoregulation, energy storage, buoyancy control and hydrodynamic efficiency [74]. However, because field metabolic rate scales with body mass, smaller cetaceans deplete their energy reserves more rapidly than large baleen whales [75,76]. In small species, a thinning blubber layer reduces insulation, requiring a higher metabolic rate to maintain body temperature and thereby accelerating blubber depletion. This creates a negative feedback loop during fasting, where increased energetic demands hasten the exhaustion of limited energy stores [77]. Because of these fundamental metabolic differences, baleen whales and killer whales are likely to experience different energetic outcomes from interference competition at herring patches.

As income breeders, killer whales have high mass-specific metabolic rates that necessitate regular prey intake to sustain their metabolism and reproduction [78]. If carousel feeding becomes less profitable owing to interference from baleen whales, killer whales may increasingly depend on alternative foraging strategies such as depredation from fisheries [42], a behavioural shift that elevates the risk of entanglement and associated mortality and human conflict [79]. Conversely, if killer whales are unable to compensate through alternative or more efficient feeding strategies, they may fail to meet their energetic requirements, leading to declines in body condition [80]. Periods of nutritional stress can trigger lipid mobilization from blubber, releasing lipophilic contaminants into circulation and vital organs, which may cause adverse health effects and increase the maternal transfer of contaminants to the foetus and nursed calves [81–83]. Winter coincides with the peak calving season for Norwegian killer whales (E. Jourdain 2014–2025, unpublished data). Pregnant and nursing animals may need up to twice their normal food intake to sustain their own metabolism plus the milk needed to sustain growth and metabolism of their calf [75,84]. As a result, any reduction in foraging efficiency during this critical time may have disproportionate impacts on maternal health and calf survival [85]. Last, chronic vessel disturbance combined with sustained interference competition can elevate physiological stress levels, with potential downstream effects on immune function, reproductive success and overall health [11,12,86–88]. Such stress-induced physiological alterations can, over time, impair individual fitness, reduce resilience to additional environmental stressors and ultimately jeopardize long-term population viability.

As capital breeders, baleen whales depend on short, productive feeding seasons in which they consume large quantities of abundant but ephemeral prey to build the energy reserves required to sustain extended fasting during migration, breeding and lactation [76]. During these migrations, mothers rely entirely on their stored blubber, using it to sustain themselves and their foetus and later to provision a growing calf, all before returning to the feeding grounds with their calf, still in a fasting state [89,90]. Therefore, the opportunity to increase prey intake at reduced energetic costs by depredating herring schools herded by killer whales, by enhancing individual body condition and energy gain, may have substantial energetic and reproductive benefits. Improved energy stores can translate into higher pregnancy rates, enhanced calf survival and shorter inter-birth intervals, as shown for humpback whales feeding on abundant Antarctic krill and other baleen whale species [88–90]. Such interactions thus highlight how changes in prey accessibility, including those mediated by interspecific foraging dynamics, can have important demographic consequences for baleen whales [75].

## 8. Conclusion

The emergence of mixed-species predator aggregations in northern Norway underscores the uneven costs and benefits of ecological change. As herring distributions have shifted, baleen whales appear to profit from *parasitizing* killer whale feeding strategies, gaining high-reward access to prey without incurring the energetic costs of prey manipulation. Killer whales, by contrast, may bear repeated energetic losses. Although the energetic consequences estimated in this study represent theoretical scenarios and remain to be empirically quantified, the relative loss in foraging efficiency compared with undisturbed periods is evident, and thus, the risk of repeated feeding disruptions during a key foraging season should not be underestimated. Winter herring are exceptionally energy-rich and likely constitute a critical resource for killer whale survival and reproduction. Therefore, reducing whale-watching pressure during this period represents a tangible management measure to help mitigate the cumulative impacts of increased competition and human disturbance.

This case study illustrates how climate- and fisheries-driven shifts in prey availability can propagate upward through food webs to reshape interactions among top predators, an underrecognized yet essential dimension of ecosystem reorganization under human influence. These cascading effects, manifesting as novel interference competition, behavioural shifts, asymmetric energetic trade-offs and intensified anthropogenic disturbance, reveal a level of ecological complexity likely more widespread than currently appreciated, though often hidden in less accessible systems. Importantly, while cumulative anthropogenic stressors are increasingly acknowledged, natural stressors such as emergent interspecific competition remain largely overlooked in such assessments. Integrating interaction-based perspectives into monitoring and management frameworks will be crucial to anticipate emerging pressures, refine conservation priorities and enhance the resilience of marine predator populations in a rapidly changing ocean.

## Supplementary Material



## Data Availability

All data are included in the paper and supplementary material. Supplementary material is available online [91].

## Theme

One contribution of 14 to a theme issue ‘Multi-predator feeding aggregations in pelagic waters’
