# Supplementary material for: Escalating competition in changing seas: baleen whales exploit killer whale prey herding
Source: Philos Trans R Soc Lond B Biol Sci. 2026 Oct 15;381(1961):20250197. doi: 10.1098/rstb.2025.0197 (PMC13678714; doi:10.1098/rstb.2025.0197)
Supplement: Supplementary file 1 [file rstb.2025.0197.zip › rstb.2025.0197/rstb20250197_si_001.pdf]

Supplementary material for:

Escalating competition in changing seas: baleen whales exploit killer whale prey herding
Eve Jourdain[1*], Chérine D. Baumgartner[2,3], David E. Cade[4], Paolo Domenici[5]

[1]Norwegian Orca Survey, Andenes, Norway
[2]Department of Environmental Systems Science, Swiss Federal Institute of Technology Zurich, Zurich, Switzerland
[3]Orcestra, Zurich, Switzerland
[4]Hopkins Marine Station, Oceans Department, Stanford University, 120 Ocean View Blvd, Pacific Grove, CA 93950, USA
[5]IBF, CNR, Institute of Biophysics, Via Moruzzi 1, 56124 Pisa, Italy
*Corresponding author

Journal: *Philosophical Transactions B*

Table S1. Data collected at herring wintering grounds between 2013 and 2024 in northern Norway. For each season (November), the following is shown: number (N) of killer whale (KW) encounters, number of killer whale carousel feeding events, number of events that involved humpback whales (HW) and fin whales (FW), number of carousel feeding events that were documented from the start - and for which the species initiating the feeding (*catalyst*) and termination of feeding immediately following baleen whale lunge(s) could be identified - number of observations of killer whales harassing humpback whales and average number of boats documented at carousel feeding events.

Season	N KW encounters	N carousel feedings	N with HW involved	N with FW involved	N documented from start	KW as catalyst	Immediate feeding termination	KW harassing HW	N boats
2013	37	22	22	0	18	17	0	0	2,5
2014	34	15	15	0	11	11	8	0	2,3
2015	107	21	16	0	7	7	4	0	2,8
2016	91	23	9	0	4	4	2	1	3,9
2017	58	2	2	0	2	2	1	0	3,5
2018	24	3	3	0	0	0	0	0	2,9
2019	66	12	6	0	1	1	3	0	2,8
2020	30	8	6	0	1	1	1	0	2,5
2021	70	3	3	0	1	1	1	0	5,2
2022	86	7	7	0	3	3	2	5	7,1
2023	88	21	18	2	15	15	10	6	7,1
2024	87	20	16	3	8	8	3	7	4
**Total**	**778**	**157**	**123**	**5**	**71**	**70**	**35**	**19**	**-**
